# SORL1-Mediated EGFR and FGFR4 Regulation Enhances Chemoresistance in Ovarian Cancer

**DOI:** 10.3390/cancers17020244

**Published:** 2025-01-13

**Authors:** Ziyan Jiang, Fangfang Bi, Zhiping Ge, Miranda Mansolf, Tobias M. P. Hartwich, Viktoriia Kolesnyk, Kevin Yang, Wonmin Park, Dongin Kim, Olga Grechukhina, Pei Hui, Sang Wun Kim, Yang Yang-Hartwich

**Affiliations:** 1 The First Affiliated Hospital of Nanjing Medical University, Nanjing 210029, China; ziyanjiang@njmu.edu.cn (Z.J.); gzp88142@163.com (Z.G.); 2 Department of Obstetrics, Gynecology, and Reproductive Sciences, Yale School of Medicine, New Haven, CT 06520, USA; biff@sj-hospital.org (F.B.); miranda.mansolf@yale.edu (M.M.); tobias.hartwich@yale.edu (T.M.P.H.); viktoriia.kolesnyk@yale.edu (V.K.); kevin.yang@yale.edu (K.Y.); wenmini@tamu.edu (W.P.); olga.grechukhina@yale.edu (O.G.); pei.hui@yale.edu (P.H.); 3 Sheng Jing Hospital of China Medical University, Shenyang 110004, China; 4 Department of Pharmaceutical Sciences, College of Pharmacy, The University of Oklahoma Health Sciences Center, Oklahoma City, OK 73117, USA; dongin-kim@ouhsc.edu; 5 Department of Pathology, Yale School of Medicine, New Haven, CT 06520, USA; 6 Department of Obstetrics and Gynecology, College of Medicine, Yonsei University, Seoul 03722, Republic of Korea; san1@yuhs.ac; 7 Yale Cancer Center, Yale School of Medicine, New Haven, CT 06520, USA

**Keywords:** SORL1, chemoresistance, EGFR, FGFR4

## Abstract

Recurrent tumors that are resistant to conventional chemotherapy are a major challenge of ovarian cancer treatment. A better understanding of the underlying molecular mechanisms of chemoresistance is critical for developing more effective targeted therapies for ovarian cancer. In this study, we analyzed the transcriptomic profiles of thirteen pairs of matching primary and recurrent ovarian cancers to identify genes that were upregulated in the recurrent tumors. Among these genes, we identified sortilin-related receptor 1 (SORL1) and its role in promoting carboplatin resistance through regulating the stability of epidermal growth factor receptor (EGFR) and fibroblast growth receptor 4 (FGFR4) using ovarian cancer models in vitro and in vivo. We further identified that an anti-SORL1 antibody inhibited the pro-tumor functions of SORL1. Our data showed that a selective inhibitor of FGFR4, FGF401, can improve the therapeutic efficacy of carboplatin in a xenograft mouse model of ovarian cancer. This study has demonstrated the therapeutic potential of targeting the SORL1/FGFR4 pathway to improve the chemoresponse of patients with recurrent and/or resistant ovarian cancer.

## Simple Summary

Treatment resistant recurrent disease is the main cause of death in patients diagnosed with ovarian cancer. The aim of this study is to better understand the mechanisms of chemoresistance, which can be targeted to improve therapeutic efficacy of current therapies in ovarian cancer. We identified *SORL1* as a gene promoting drug resistance in recurrent ovarian cancer. We demonstrated the molecular mechanism by which SORL1 enhances cancer cell proliferation and platinum resistance through stabilizing the receptors of several growth factors. Therapeutic agents targeting SORL1 and its related pathways have potential to overcome the platinum resistance of recurrent ovarian cancer.

## 1. Introduction

Ovarian cancer causes more deaths than any other gynecological cancer. The main cause of mortality in patients with ovarian cancer is the recurrent diseases that are resistant to currently available treatments. Current standard treatment for ovarian cancer involves debulking surgery and taxane–platinum combination therapy. The resistance to taxane- or platinum-based agents involves a plethora of complex molecular pathways. The identification of the key molecules in these pathways is a critical step towards developing targeted therapies to improve the clinical outcomes of patients with recurrent ovarian cancer.

In this study, we have identified that the upregulation of sortilin-related receptor 1 (SORL1) is associated with recurrent ovarian cancer and resistance to carboplatin. SORL1, also known as SORLA or LR11, is an intracellular sorting receptor. It was first discovered that SORL1 contributes to endosomal degradation and recycling pathways in neurons [1,2,3]. *SORL1* mutation can cause endosome defects leading to dysfunction in the trafficking and recycling of amyloid precursor protein. Therefore, *SORL1* mutation was associated with Alzheimer’s disease [4,5,6].

The role of SORL1 in human cancer has not been extensively studied. It was first demonstrated in breast cancer models that SORL1 could bind to human epidermal growth factor receptor 2 (HER2) in the plasma membrane and intracellular vesicles, which regulates the endosomal trafficking and subcellular distribution of HER2 [7]. The increased expression of SORL1 promotes HER2 recycling to the cell surface of breast cancer cells. Due to the critical role of HER2 in breast cancer tumor progression and response to treatment, the inhibition or depletion of SORL1 is a potential therapeutic strategy for increasing the drug sensitivity of breast cancer cells [8].

In ovarian cancer, one study reported that SORL1 can stabilize ATP-binding cassette subfamily member 1 (ABCB1, also known as multidrug resistance protein 1 or MDR1) to enhance resistance to cisplatin [9]. Even though the two cell lines (A2780 and SKOV3) used in this previous study do not represent the most common subtype of ovarian cancer, high-grade serous carcinoma [10,11], this study supports the potential role of SORL1 in contributing to chemoresistance in ovarian cancer. In our study, we further investigated the mechanisms underlying the potential role of SORL1 in promoting ovarian cancer cell growth and platinum resistance. Based on our data that SORL1 was upregulated in recurrent tumors, and that it promoted carboplatin resistance and the expression levels of epidermal growth factor receptor (EGFR) and fibroblast growth factor receptor 4 (FGFR4), we further hypothesize that SORL1 regulates the endosomal trafficking and stability of EGFR and FGFR4 to promote the platinum resistance of recurrent ovarian cancer. EGFR (or HER1) belongs to the epidermal growth factor receptor family that also includes HER2, HER3, and HER4. They are receptor tyrosine kinases and form homodimers or heterodimers upon ligand binding. These members are frequently overexpressed in a variety of cancer. EGFR overexpression is associated with poor prognosis in ovarian cancer [12]. Clinical trials of EGFR-targeted tyrosine kinase-based inhibitors (TKIs) have not achieved significant clinical benefit in ovarian cancer. Therefore, new approaches to stratify patients and a better understanding of the dysregulated EGFR pathway in ovarian cancer will be critical for improving the efficacy of EGFR-targeted therapy. The aberrant FGFR4 pathway was identified in human cancers, which can be caused by gene amplification, post-transcriptional dysregulation, mutations, or alternative splicing of the *FGFR4* gene, and the overexpression of its ligands in cancer or stromal cells [13,14,15,16]. In ovarian cancer, the overexpression of FGFR4 was significantly associated with decreased overall survival duration [17]. High expression levels of FGFR4 and FGF19, which is one of the ligands with the highest affinity and specificity to FGFR4, together can provide an even better prognostic marker for advanced stage high-grade serous ovarian carcinoma [18]. The inhibition or silencing of FGFR4 was shown to suppress tumor progression in preclinical ovarian cancer models in vitro and in vivo [17]. The therapeutic potential of FGFR4 selective inhibitors has not been investigated in ovarian cancer models.

In this study, we identified the upregulation of SORL1 in recurrent ovarian cancer and demonstrated SORL1’s role in promoting carboplatin resistance using a common ovarian cancer cell line, OVCAR8, and a patient-derived high-grade serous ovarian cancer cell line, KRCH31. Our data also suggest that SORL1 interacts with EGFR and FGFR4, and their interactions are required to maintain the high levels of EGFR and FGFR4 proteins in ovarian cancer cells. This study also demonstrated the potential of targeting SORL1 with an antibody blocker or targeting FGFR4 with a small molecule inhibitor as new strategies to improve the response to chemotherapy in patients with recurrent and resistant ovarian cancer.

## 2. Materials and Methods

### 2.1. Cell Lines and Reagents

OVCAR8 cells were obtained from Dr. Anirban Mitra, Indiana University School of Medicine [19,20]. A2780 cells and patient-derived ovarian cancer cell lines R182 and R127 were provided by Dr. Gil Mor [21], and the patient-derived high-grade serous ovarian cancer cell line KRCH31 was provided by Dr. Alessandro Santin, Yale University School of Medicine. This cell line was derived from the tumor sample of a patient with recurrent chemoresistant ovarian cancer [22]. Ovarian cancer cell lines were propagated in RPMI 1640 medium containing 10% fetal bovine serum (FBS), L-glutamine, and penicillin/streptomycin. They were all cultured in incubator at 37 °C (5% CO_2_). The SORL1 plasmid was provided by Dr. Stephen Strittmatter, Yale University School of Medicine [23]. The cytokines and growth factors were purchased from PeproTech (Cranbury, NJ, USA), including interleukin 6 (IL-6), vascular endothelial growth factor (VEGF), transforming growth factor beta (TGF-β), intercellular adhesion molecule 1 (ICAM-1), C-X-C motif chemokine 10 (CXCL10), C-X-C motif chemokine 11 (CXCL11), C-X-C motif chemokine 12 (CXCL12), brain-derived neurotrophic factor (BDNF), fibroblast growth factor acidic (FGF-acidic or FGF-1), and epidermal growth factor (EGF). Carboplatin, fisogatinib (BLU-554), and FGF401 were purchased from Cayman Chemical (Ann Arbor, MI, USA). In the 3D model, one thousand cancer cells were cultured in each droplet as previously described [24].

### 2.2. Clinical Sample Collection and Human Transcriptomic Array (HTA)

Tumors were collected and fixed to prepare the formalin-fixed paraffin-embedded (FFPE) tissue samples. FFPE sections (10–20 μm thickness, three pieces per sample) were used to extract total RNA with the FFPE RNA/DNA Purification Plus Kit (Norgen Biotek, Thorold, ON, Canada). RNA samples with DV200 > 30% and yield of >100 ng (Supplemental Table S1) were used for microarray analysis. The Affymetrix GeneChip HTA 2.0 microarray (Thermo Fisher Scientific, Waltham, MA, USA) was used for the analysis and results were visualized using Transcriptome Analysis Console (TAC) 4.0.2. Study approval was obtained from the Institutional Review Board at Yonsei University and Yale University (see details of the approval in the Institutional Review Board Statement section).

### 2.3. Western Blot

Cells were lysed using cell lysis buffer (1% Triton X-100, 0.05% SDS, 100 mM Na_2_HPO4, and 150 mM NaCl). About 15–20 μg of each protein lysate were electrophoresed on a 12% SDS-polyacrylamide gel and transferred onto Amersham Hybond 0.45 PVDF membranes (GE Healthcare, Chicago, IL, USA). After blocking with 5% non-fat milk in PBS-0.05% Tween 20, the membranes were incubated with primary antibodies at 4 °C overnight, and then secondary antibodies for 1 h at room temperature. The blots were developed using Clarity or Clarity Max Western ECL Blotting Substrates (Bio-Rad, Hercules, CA, USA). Antibody information is listed in Supplemental Table S2.

### 2.4. RNA Extraction and Quantitative Real-Time PCR (QPCR)

Total RNA was extracted using Total RNA Purification Kit (Norgen Biotek) according to the manufacturer’s instruction. cDNA was synthesized with qScript cDNA SuperMix Kit (Quantabio, Beverly, MA, USA). QPCR was performed using SYBR Green Supermix (Bio-Rad, Hercules, CA, USA) followed by detection with the CFX Connect QPCR detection system (Bio-Rad). GAPDH was used as a reference gene. Relative expression was calculated using the comparative ΔΔCT method. All reactions were performed with 5 biological replicates. And each replicate included 3 technical replicates. Primer sequences are provided in Supplemental Table S3.

### 2.5. Transient Transfection and Stable Knockdown of SORL1 Expression in Ovarian Cancer Cell Lines

To overexpress SORL1 in ovarian cancer cells, cells were transfected with the SORL1-containing plasmid [23] using ViaFect Transfection Reagent (Promega, Madison, WI, USA) according to the manufacturer’s instructions. The control cells were transfected with an empty vector. To transiently knockdown SORL1 expression, siRNA was transfected using Lipofectamine RNAiMAX (Life Technologies, Carlsbad, CA, USA). siRNA and the RNAiMAX reagent were diluted in Opti-MEM I Reduced Serum Medium (Life Technologies, Carlsbad, CA, USA) separately before being mixed by vortexing. The mix was added to the cells and incubated for 48 h in the incubator. siRNAs and the control were purchased from Millipore-Sigma (The Woodlands, TX, USA). They are validated negative control siRNA (SIC001), and esiRNAs targeting *SORL1* (gene: *SORLA*, EHU003061). To establish the stable SORL1 knockdown cell lines, lentiviral shRNA targeting SORL1 and control virus particles (sc-44375-V and sc-108080, Santa Cruz Biotechnology, Dallas, TX, USA) were used to infect the ovarian cancer cell lines. These virus particles contain three to five expression constructs, and each construct encodes a SORL1-specific shRNA. Puromycin (1 μg/mL) was added to the culture medium to select the positive cells. Information about the siRNA, shRNA, and plasmid is provided in the Supplemental Table S4.

### 2.6. Cell Viability, Apoptosis (Caspase-3/7 Activity), and Cell Cycle Analyses

Cells were plated at a concentration of 6 × 10^3^ cells/well in 96-well plates. Twenty-four hours later they were treated with the desired concentrations of carboplatin or anti-SORL1 antibody for 48 h. Then, CellTiter-Glo cell viability reagent (Promega) was added to each well and incubated for 10 min at 37 °C. The CellTiter-Glo luminescent signal was detected using the GloMax Navigator Microplate Luminometer (Promega, Madison, WI, USA).

Caspase-3 activity was evaluated using the Caspase-Glo 3/7 Assay kit (Promega). Protein lysate (10 μg) was diluted to a final volume of 50 μL. An equal volume of the Caspase-Glo 3/7 Reagent was added to the lysate and incubated at room temperature for 1 h before they were recorded using a Navigator Microplate Luminometer.

For cell cycle analysis, cells were collected and washed with ice-cold PBS, and then fixed in ice-cold 70% ethanol at −20 °C for 30 min. After the cells were washed in PBS, they were incubated with ribonuclease A (100 μg /mL) for 5 min at room temperature and stained with propidium iodide (20 μg/mL) in PBS. Cell cycle phase distributions were analyzed with Flow-Jo v. 8.7.

### 2.7. Co-Immunoprecipitation (Co-IP)

Co-IP was conducted using SureBeads Protein G Magnetic Beads (Bio-Rad, Hercules, CA, USA). Briefly, antibodies (1 μg) were incubated with the beads (100 μL) for 10 min at room temperature. Cell lysate (500 μL, 1 μg/ μL) was incubated with the beads for one hour at room temperature on a rotating mixer. The beads were washed three times with PBST. The IP products were eluted using 40 μL 1× Laemmli buffer by incubating at 70 °C for 10 min. Western blot was performed to analyze the IP products.

### 2.8. Proximity Ligation Assay (PLA)

Cells were cultured on chamber slides, fixed with 4% paraformaldehyde in PBS for 10 min at room temperature, and then washed with PBS three times followed by incubation in a blocking buffer. PLA was performed using the Duolink In Situ Red Starter Kit (#DUO92101, Sigma-Aldrich, St. Louis, MO, USA). Briefly, mouse anti-SORL1 and rabbit anti-EGFR or rabbit anti-FGFR4 antibodies were diluted in antibody diluent and incubated with the samples at 4 °C overnight. After three times washing with 1× PBS, PLA probes were diluted 1:5 and incubated with samples for 60 min at 37 °C. After washing with 1× wash buffer A twice, the ligation mix was incubated for 30 min at 37 °C. After washing with 1× wash buffer A twice, the amplification mix was added to incubate for 100 min at 37 °C. The slides were washed with 1× wash buffer B twice and 0.01× wash buffer B for 1 min. Duolink In Situ Mounting Medium with 4,6-diamidino-2-phenylindole was used to mount the samples for 15 min. The slides were imaged using a confocal microscope (Leica TCS SP8; Leica Microsystems, Wetzlar, Germany).

### 2.9. Animal Models

Ovarian cancer cell lines KRCH31 (7 × 10^6^ cells/injection) were injected subcutaneously into the dorsal region of anesthetized nude mice (5 mice/group). When a tumor was formed, the tumor’s diameter was recorded every three days for three to five weeks. When the tumor volume exceeded 500 mm^3^, the mice were euthanized, and xenografts were collected. All animal experiments were approved by the Yale University Institutional Animal Care and Use Committee. Carboplatin (5 mg/kg) and/or FGF401 (3 mg/kg) were intraperitoneally injected weekly.

### 2.10. Statistical Analysis

Excel Version 16.92 and Prism Version 10.0.3 (GraphPad Software, La Jolla, CA, USA) were used to perform statistical analyses. Statistical differences between two groups were compared using Student’s *t*-test. A two-way analysis of variance (ANOVA) test (Tukey’s multiple comparison test) was used to test hypotheses about effects in multiple groups with two variables. * *p*  <  0.05 was considered significant if not specified. The RNA-sequencing data of SORL1 was obtained from the Cancer Genome Atlas (TCGA) dataset of ovarian serous cystadenocarcinoma (see details of the TCGA data in the Results Section). The patient survival dataset was available from https://v22.proteinatlas.org/. Kaplan–Meier analysis was used for the survival analysis. The results of the statistical analysis were indicated in the figures.

## 3. Results

### 3.1. SORL1 Is Upregulated in Recurrent Tumors in Ovarian Cancer

**Figure 1:**
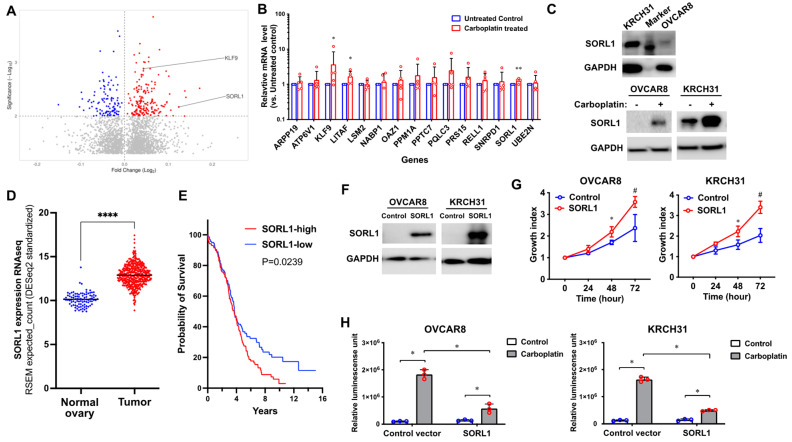
The increased expression of SORL1 is associated with the resistance to carboplatin in ovarian cancer. (**A**) A volcano plot of the data generated from the Human Transcriptome Array of the paired recurrent tumors versus primary ovarian cancers. n = 15. The genes of which expression was downregulated in the recurrent tumors (*p* < 0.01 and 0 < fold change < 1) were labeled as blue dots. The genes of which expression was upregulated (*p* < 0.01 and fold change > 1) were labeled as red dots. The gray dots indicate genes of which expression was upregulated or downregulated, but the *p*-value was larger than the cutoff. The genes of which expression was not changed in the recurrent tumors versus the primary tumors (fold change = 1) were not shown in this graph. (**B**) QPCR of the target genes in five ovarian cancer cell lines (OVCAR8, A2780, KRCH31, R127, and R1820) that were untreated or treated with 100 μM carboplatin for 48 h and recovered for 24 h. * *p* < 0.05, ** *p* < 0.005. (**C**) A western blot of SORL1 protein in ovarian cancer cell lines that were untreated or treated with 100 μM carboplatin for 48 h and recovered for 24 h. (**D**) RNA-sequencing data of SORL1 in ovarian cancer samples (n = 418, TCGA dataset) and normal ovary (n = 93, the GTEx dataset). **** *p* < 0.0001. (**E**) Kaplan–Meier curves show the comparison of patient survival between SORL1-high (n = 209) and SOLR-low (n = 164) cases of ovarian cancer in the TCGA dataset. (**F**) A western blot of SORL1 protein in ovarian cancer cell lines transfected with SORL1 or control plasmids. (**G**) The cell growth curves of ovarian cancer cell lines overexpressing SORL1 or control plasmids. * *p* < 0.05, # *p* < 0.001. (**H**) Caspase-3/7 activity of ovarian cancer cell lines transfected with SORL1 or control plasmids after treatment of carboplatin for 48 h. * *p* < 0.05.

To identify genes that are associated with recurrent ovarian cancer, we collected primary tumors and their matched recurrent tumors from fifteen patients diagnosed with high-grade serous ovarian cancer. Their basic demographics are included in Supplemental Table S5. HTA analysis of the RNA samples extracted from the tumor FFPE sections revealed 436 genes (312 coding and 114 non-coding genes, *p* < 0.01) that were differentially expressed in recurrent tumors in comparison to their primary tumors (Figure 1A).

### 3.2. SORL1 Protein Level Is Increased in Ovarian Cancer Cells That Survived Carboplatin Treatment

To validate whether the identified genes are related to chemoresistance, we compared their expression in carboplatin-treated and untreated ovarian cancer cells. We treated five ovarian cancer cell lines (OVCAR8, A2780, KRCH31, R127, and R182) in vitro with 100 μM carboplatin for 48 h and allowed the surviving cells to recuperate for 24 h. Using QPCR assay, we compared the expression levels of the identified genes between the cancer cells that survived carboplatin treatment and the untreated control cells. As shown in the representative data in Figure 1B, the surviving cancer cells had an increased expression of KLF9 and SORL1, which are statistically significant. The carboplatin-induced expression of SORL1 mRNA was subtle (i.e., 28 ± 8% increase). However, induction at the protein level was more significant (Figure 1C). We first validated the basal expression of SORL1 protein in OVCAR8 and KRCH31 cells (Figure 1C). We then confirmed that the OVCAR8 and KRCH31 cells that survived carboplatin treatment show a significantly higher level of SORL1 protein in comparison to the untreated control cells (Figure 1C).

### 3.3. SORL1 Expression Is Upregulated in Ovarian Cancer in Comparison to the Normal Ovary

Since very limited data have been reported regarding the expression of SORL1 in ovarian cancer, to set up a foundation for our further research into SORL1 functions in ovarian cancer tumorigenesis and chemoresponse, we first analyzed the TCGA dataset of high-grade serous ovarian cancer and normal ovary samples. We found that SORL1 expression is significantly higher in the tumor samples compared to the normal ovary samples (Figure 1D). This result suggests that the abnormal overexpression of SORL1 has the potential to benefit the survival of cancer cells and the development of ovarian cancer.

### 3.4. A Higher Level of SORL1 Expression Is Associated with Shorter Survival in Ovarian Cancer

Moreover, we analyzed the SORL1 RNA expression and patient survival data using the TCGA high-grade serous ovarian cancer cases (Supplemental Table S6). Based on the expression levels of SORL1, patients were divided into two groups, in which the patients with higher levels of SORL1 expression showed shorter overall survival (Figure 1E). This observation supports the possibility that SORL1 plays a role in promoting tumor progression. To date, we found only one study on SORL1 in ovarian cancer [9]. Therefore, we decided to conduct mechanistic studies to better understand the role of SORL1 in regulating the growth of ovarian cancer cells and their response to chemotherapy.

### 3.5. Upregulated Expression of SORL1 Promotes Cell Proliferation and Resistance to Carboplatin Treatment in Ovarian Cancer Cell Lines

To better understand the function of SORL1 in ovarian cancer, we overexpressed SORL1 in ovarian cancer cell lines OVCAR8 and KRCH31 using transient transfection (Figure 1F). The ovarian cancer cells overexpressing SORL1 showed increased proliferation in comparison to the control cells (Figure 1G). SORL1 overexpression also inhibited carboplatin-induced apoptosis, which was demonstrated by the significantly reduced caspase-3 activity in the carboplatin-treated cells overexpressing SORL1 in comparison to the control cells treated with carboplatin (Figure 1H).

### 3.6. SORL1 Knockdown Inhibits Cell Proliferation and Improves Sensitivity to Carboplatin-Induced Apoptosis in Ovarian Cancer Cell Lines

**Figure 2:**
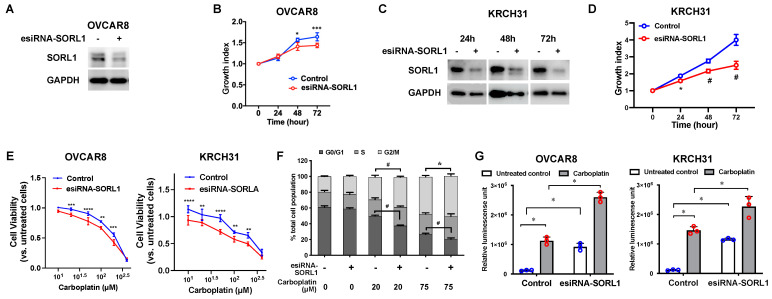
SORL1 knockdown inhibits cell proliferation and improves sensitivity of ovarian cancer cell lines to carboplatin. (**A**) Western blot of SORL1 protein in OVCAR8 ovarian cancer cells transfected with control esiRNA (control) or SORL1-targeting esiRNAs (esiRNA-SORL1) at 48 h post transfection. (**B**) Cell growth curves of OVCAR8 cells. (**C**) Western blot of SORL1 protein in KRCH31 cells transfected with control or SORL1-targeting esiRNAs. (**D**) Cell growth curves of KRCH31 cells. (**E**) Cell growth curves of OVCAR8 and KRCH31 cells treated with carboplatin. (**F**) Distribution of OVCAR8 cells in different phases of cell cycle. (**G**) Caspase-3/7 activity of OVCAR8 and KRCH31 cells treated with 100 μM carboplatin. Two-way ANOVA, * *p* < 0.05, ** *p* < 0.005, *** *p* < 0.0005, **** *p* < 0.0001, # *p* < 0.0001.

When we knocked down SORL1 expression in OVCAR8 cells (Figure 2A), their proliferation was inhibited (Figure 2B). Cell proliferation was also inhibited in KRCH31 cells when we knocked down SORL1 (Figure 2C,D). SORL1 knockdown also increased the sensitivity to carboplatin treatment in these two cell lines (Figure 2E). The IC50 values of carboplatin were decreased in OVCAR8 cells (from 204.8 μM to 126.4 μM) and KRCH31 cells (from 260.9 μM to 178.9 μM). Carboplatin treatment in OVCAR8 cells induced cell cycle arrest at the G2/M phase due to unrepairable DNA damage (Figure 2F). Under carboplatin treatment, compared to the control cells, the cancer cells with SORL1 knockdown contained a higher percentage of cells in the G2/M phase. This observation again supports that the downregulation of SORL1 made cancer cells more vulnerable to carboplatin treatment. SORL1 knockdown increased carboplatin induced-apoptosis, which was demonstrated by the increased caspase-3 activity (Figure 2G).

### 3.7. SORL1 Regulates EGF and FGF Signaling Through Interactions with EGFR and FGFR4 in Ovarian Cancer Cell Lines

**Figure 3:**
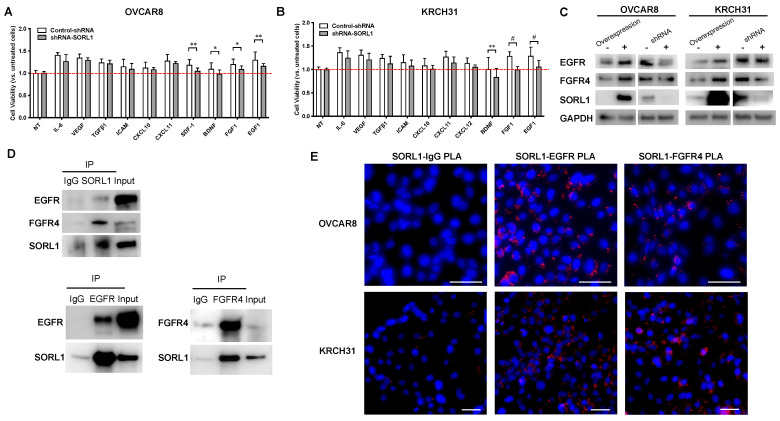
SORL1 is involved in regulation of EGFR and FGFR4 signaling. (**A**,**B**) Cell viability of OVCAR8 (**A**) or KRCH31 (**B**) cells treated with ten different cytokines or growth factors. OVCAR8 or KRCH31 cells stably expressed control shRNA (control-shRNA) or shRNA targeting SORL1 (shRNA-SORL1). NT, untreated control. Red dotted lines indicate levels of cell viability in NT groups. Two-way ANOVA, * *p* < 0.05, ** *p* < 0.01, # *p* < 0.001. (**C**) Western blot images of OVCAR8 and KRCH31 cells with overexpression of knockdown (shRNA) of SORL1. (**D**) Co-immunoprecipitation (co-IP) of SORL1 and EGFR or FGFR4 using protein lysate of KRCH31 cells. (**E**) Proximity Ligation Assay (PLA) of SORL1 and EGFR or FGFR4. Scale bar = 50 μm.

We designed a screening experiment in which the control and SORL1 knockdown cell lines were treated with ten growth factors that were associated with chemoresistance in ovarian cancer. By comparing the effects of growth factor treatment on the control and SORL1 knockdown cell lines, we identified that SORL1 was involved in the cancer cell growth stimulated by CXCL12, BDNF, FGF1, and EGF1 in OVCAR8 cells (Figure 3A) and by BDNF, FGF1, and EGF1 in KRCH31 cells (Figure 3B). We hypothesize that SORL1 mediates FGF1 and EGF1 signaling through regulating the expression levels of the receptors for FGF1 and EGF1. Using antibodies against several known receptors of FGF1 and EGF1 in the western blot screening and co-immunoprecipitation (co-IP), we identified that the levels of EGFR and FGFR4 proteins were affected by SORL1. Our data showed that the overexpression of SORL1 caused the upregulation of EGFR and FGFR4 proteins, whereas SORL1 knockdown downregulated EGFR and FGFR4 (Figure 3C). The result of co-IP in the KRCH31 cells demonstrates that SORL1 interacts with EGFR and FGFR4 (Figure 3D). Using Proximity Ligation Assay (PLA), we also validated the SORL1-EGFR and SORL1-FGFR4 interactions in situ (Figure 3E). These results provide evidence that SORL1 can regulate EGF1 and FGF1 signaling in ovarian cancer possibly through its interaction with EGFR and FGFR4.

### 3.8. Anti-SORL1 Antibody Reduces Viability of Ovarian Cancer Cell Lines and Improves Chemosensitivity

**Figure 4:**
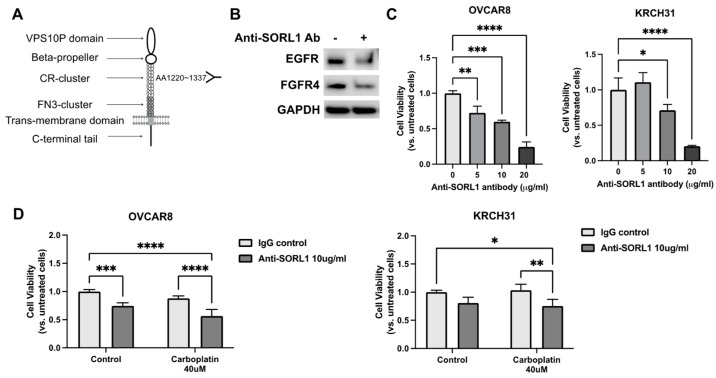
Treatment with anti-SORL1 antibody reduces viability of ovarian cancer cells. (**A**) Schematic description of domain in SORL1 protein and targeting region of anti-SORL1 antibody (AA1220-1337). (**B**) Western blot of OVCAR8 cells treated with anti-SORL1 antibody (Ab) in comparison to untreated control. (**C**) Cell viability of OVCAR8 and KRCH31 cells treated with different concentrations of anti-SORL1 Ab. One-way ANOVA, * *p* < 0.05, ** *p* < 0.01, *** *p* < 0.005, **** *p* < 0.001. (**D**) Cell viability of OVCAR8 and KRCH31 cells treated with anti-SORL1 Ab (10 μg/mL) in combination with carboplatin (40 μM). Two-way ANOVA, * *p* < 0.05, ** *p* < 0.01, *** *p* < 0.005, **** *p* < 0.001.

It was shown that the extracellular domain of SORL1 interacts directly with HER2 and HER3, and an antibody targeting the extracellular domain of SORL1 (Figure 4A) can disrupt its ability to support HER2/HER3 trafficking in breast cancer cells [7,8].

In the ovarian cancer cell line OVCAR8, we demonstrated that treatment with this antibody at 10 μg/mL for 24 h downregulated the levels of EGFR and FGFR4 (Figure 4B). Moreover, when ovarian cancer cells were cultured in a 3D culture model, the single treatment of this SORL1-blocking antibody reduced the cell viability in a dose-dependent manner (Figure 4C). Treatment with the anti-SORL1 antibody at a concentration of 20 μg/mL for 48 h reduced the viability of OVCAR8 cells to 24 ± 6.9% and KRCH31 cells to 20 ± 1.2% in comparison to their untreated control groups (Figure 4C). A combination treatment of 10 μg/mL antibody and 40 μM carboplatin achieved a better therapeutic effect than the additive effects of the two treatments individually (Figure 4D). In the OVCAR8 cells, the combination reduced cell viability by about 44%, while the antibody alone reduced cell viability by 25% and carboplatin alone reduced cell viability by 12%. In the KRCH31 cells, the combination reduced cell viability by about 24%, while the antibody alone reduced cell viability by 19% and carboplatin alone showed no effect.

### 3.9. SORL1 Knockdown Inhibits Tumor Growth in Xenograft Mouse Model of Ovarian Cancer and Downregulates EGFR and FGFR4

**Figure 5:**
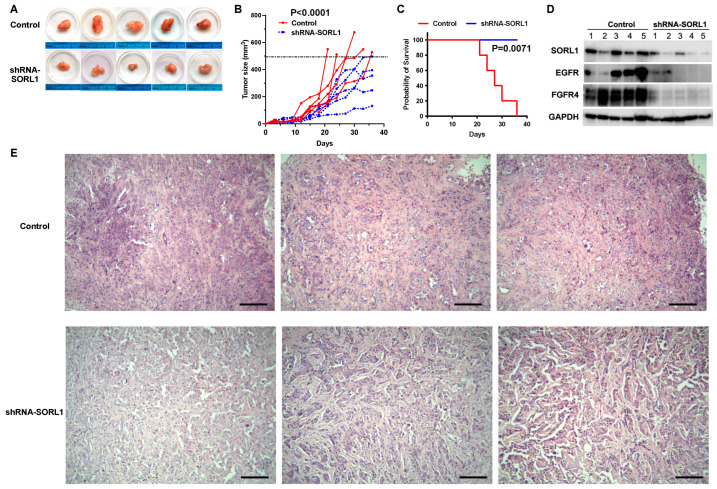
SORL1 knockdown in ovarian cancer cells inhibits tumor growth in vivo. (**A**) Images of tumors dissected from mice injected with KRCH31 cells expressing lentiviral control vector or lentiviral shRNA targeting SORL1 (shRNA-SORL1). (**B**) Tumor growth curves (n = 5 mice/group). Two-way ANOVA. *p* < 0.001. (**C**) Survival curves of mice. (**D**) Western blot of tumor samples collected from mice. (**E**) Representative images of H&E staining using tumors from mice. Scale bar = 100 μm.

The patient-derived KRCH31 cell line can form subcutaneous tumors in the immune-compromised mice as a xenograft model of ovarian cancer [22]. When we knocked down SORL1 expression in the KRCH31 cells by stably expressing SORL1-targeting shRNA, tumor growth was significantly suppressed in comparison to the tumors formed by the control KRCH31 cells (Figure 5A,B). The inhibition of SORL1 significantly improved the survival of animals in this experiment (Figure 5C). When we collected the tumors and examined the levels of protein expression, we found that SORL1 knockdown led to the downregulation of both EGFR and FGFR4 in the tumor samples (Figure 5D). Through H&E staining of the tumor tissue sections, we identified that tumors with SORL1 knockdown showed increased amounts of necrosis in comparison to the control tumors (Figure 5E). Compared to the control tumors, the SORL1 knockdown tumor tissues showed significant discoloration and loss of cellular details. Widespread gaps were readily apparent in the SORL1 knockdown tumor tissues, which indicates the presence of fluid and exudative material within the tumors. These are common morphological features of necrotic tissues.

### 3.10. Treatment with FGF401, FGFR4-Specific Inhibitor, Enhances Sensitivity to Carboplatin of SORL1-Expressing Ovarian Cancer in Xenograft Mouse Model

**Figure 6:**
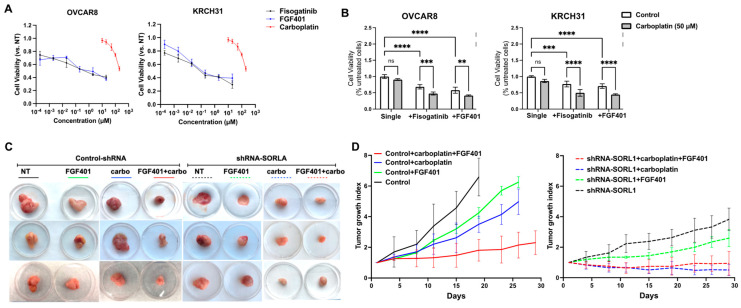
FGFR4 inhibitors have potential to increase sensitivity to carboplatin in ovarian cancer cells. (**A**) Cell viability of OVCAR8 and KRCH31 cells treated with carboplatin or FGFR4 inhibitors (FGF401 and fisogatinib). (**B**) Cell viability of OVCAR8 and KRCH31 cells treated with combination or single treatment of carboplatin (50 μM) and/or FGFR4 inhibitors. Two-way ANOVA, ** *p* < 0.01, *** *p* < 0.005, **** *p* < 0.001. ns, not significant. (**C**) Representative images of tumors collected from mice treated with combination or single treatment of carboplatin (5 mg/kg, IP) and/or FGF401 (3 mg/kg, IP). (**D**) Tumor growth curves. (n = 5 mice/group).

Clinical trials of EGFR-targeted tyrosine kinase-based inhibitors have not achieved significant benefits in patients with persistent or recurrent ovarian cancers [25,26,27,28,29]. Therefore, we propose to further explore the potential of FGFR4 inhibitors in targeting recurrent ovarian cancers. To better assess the tumor-suppressing potential specifically due to the inhibition of FGFR4, we evaluated the effect of small molecular FGFR4 inhibitors on ovarian cancer cell lines in vitro and in vivo. Treatments with FGFR4 inhibitors, FGF401 and fisogatinib, both decreased the viability of OVCAR8 and KRCH31 cells with lower EC50 values compared to carboplatin (Figure 6A). When combined with carboplatin, the FGF401 or fisogatinib treatments further increased the cytotoxicity of carboplatin in ovarian cancer cell lines (Figure 6B). When FGF401 was administered as a single agent or in combination with carboplatin in the mouse xenograft model formed by the subcutaneous injection of KRCH31 cells, the growth of tumors in mice treated with the combination therapy was suppressed more effectively in comparison to the other groups (Figure 6C,D). FGF401 also showed moderate suppressive effects on the tumors formed by KRCH31 cells with SORL1 knockdown (Figure 6C,D). However, FGF401 did not significantly improve the effect of carboplatin since carboplatin was already very effective on the tumors with the knockdown of SORL1.

## 4. Discussion

When ovarian cancers recur, they are usually widespread in the peritoneal cavity and have developed resistance to currently available therapies. Patients deteriorate very quickly after the recurrent tumors are diagnosed. Clinical studies also have shown that secondary surgery to remove the recurrent tumors followed by chemotherapy did not result in longer overall survival than chemotherapy alone [30]. This is not only a major clinical challenge but also makes it challenging to obtain recurrent tumor samples for research. However, a better understanding of the molecular mechanisms underlying chemoresistance is a critical step towards developing more effective treatments for patients with recurrent resistant ovarian cancer. Therefore, in this study, we attempted to dissect the gene expression difference between the recurrent tumors and primary ovarian cancers through a small cohort of paired primary and recurrent tumor samples.

We first examined the transcriptomic profiles of patients’ primary and recurrent ovarian cancers and identified the upregulation of SORL1 in recurrent tumors. Many other genes were also identified as differentially expressed when tumors recurred. These genes are currently under further validation or functional investigation. *SORL1* is unlikely to be the most important gene and definitely not the only gene that regulates tumor progression and chemoresponse in ovarian cancer. We focused on SORL1 in this study to provide more evidence on the role of SORL1 in ovarian cancer and carboplatin resistance since very limited knowledge related to SORL1 in ovarian cancer was developed. Our findings also suggest that the interaction of SORL1 with EGFR and FGFR4 may play a role in increasing the levels of EGFR and FGFR4 proteins in ovarian cancer cells. As prior research suggested, the inhibition or downregulation of EGFR or FGFR4 can lead to tumor suppression. Our study also demonstrated that an antibody targeting the extracellular domain of the SORL1 protein could inhibit the function of SORL1 leading to the downregulation of EGFR and FGFR4. Our findings suggest that the anti-SORL1 antibody and a small molecule inhibitor specific to FGFR4 both have the potential to overcome carboplatin resistance in ovarian cancer cells.

Ovarian cancers are heterogeneous and consist of several distinct histotypes, including high-grade serous, low-grade serous, clear cell, endometrioid, and mucinous types. High-grade serous ovarian carcinoma is the most common type. Up to 90% of the advanced stage ovarian cancer cases are high-grade serous type. In this study, the primary and recurrent tumor samples were collected from patients who were diagnosed with high-grade serous ovarian cancer. We also analyzed copy number variants (CNVs) of these tumor samples using the OncoScan CNV Assay, which confirmed that these tumors possessed high levels of copy number aberrance as a genetic signature of high-grade serous ovarian cancer. We used two cell lines for the in vitro and in vivo mechanistic studies. The OVCAR8 cell line was identified as a high-grade serous ovarian cancer cell line with resistance to carboplatin [31,32]. The KRCH31 cell line was derived from a patient who was diagnosed with high-grade serous ovarian tumor and developed resistance to platinum–paclitaxel combination therapy [22]. When the KRCH31 cells are injected into immune deficient mice, they can form tumors resembling the human high-grade serous carcinoma. These models allow for our investigation of SORL1 functions in carboplatin-resistant high-grade serous ovarian cancer. One of the limitations of using these models is the deficient immune system. Immune cells play critical roles in regulating ovarian cancer progression and treatment responses. To better understand the mechanism of SORL1 and its potential as a therapeutic target, we still need to fill the knowledge gap regarding the involvement of immune cells. Another limitation of our in vivo model is that subcutaneous xenografts are not ideal for recapitulating the tumor microenvironment of ovarian cancer in the peritoneal cavity. These aspects will be addressed in our future studies using syngeneic mouse models with normal immune systems through intraperitoneal injections (or intrabursal injections to the ovaries) of cancer cells.

In ovarian cancer, EGFR overexpression is associated with poor prognosis and decreased responsiveness to chemotherapy [12]. This observation led to several clinical trials of EGFR-targeted tyrosine kinase-based inhibitors. These trials in unselected ovarian cancer patients only achieved a 0–6% response rate in patients with persistent or recurrent tumors and did not show survival benefit as a maintenance treatment for patients with stable disease after first-line chemotherapy [25,26,27,28,29]. Therefore, new approaches to stratify patients and a better understanding of the dysregulation in the EGFR pathway will be critical for improving the efficacy of EGFR-targeted therapy in ovarian cancer. Our results provide new mechanistic insights regarding the regulation of EGFR by SORL1 in ovarian cancer.

Due to the overexpression of FGFR4 in ovarian cancer and its role in promoting cancer cell proliferation, FGFR4 inhibitors have therapeutic potential in ovarian cancer [17,18]. In our study, we focused on the FGFR4 selective inhibitors, which selectively inhibit FGFR4 and do not inhibit (or have significantly lower activity on) FGFR1, FGFR2, or FGFR3. Previous studies have tested FGFR1/2/3 inhibitors AZD454 and CPL304-110-01 in ovarian cancer cell lines, which demonstrated the therapeutic potential of these inhibitors [33,34]. FGF401 (official name, roblitinib) is a highly selective potent FGFR4 inhibitor. It has demonstrated antitumor activity in hepatocellular carcinoma models [35]. In the first-in human phase 1/2 trial, FGF401 treatment alone was safe and showed preliminary clinical efficacy in patients with hepatocellular carcinoma [36]. Our study provides evidence that FGF401 can target the patient-derived chemoresistant ovarian cancer cells and sensitize them to carboplatin treatment in the in vitro and xenograft models. Our findings support the potential of FGFR4 inhibitors as new therapeutic agents for ovarian cancer.

Currently, small molecule inhibitors of SORL1 have not been developed. The SORL1-blocking antibody binds to the extracellular domain of SORL1, which allows for the inhibition of SORL1 functions in endosomal trafficking. Our results and previous studies using this antibody in breast cancer models have demonstrated the feasibility of targeting SORL1 to overcome drug resistance in ovarian and breast cancers. However, to inhibit SORL1 in cancer cells more effectively and more specifically in a clinical setting, we still need to overcome several critical barriers. For example, first, the in vivo stability and bioavailability of the anti-SORL1 antibody may limit the therapeutic efficacy. Second, SORL1 is a key regulator of endosomal trafficking in neurons. SORL1 RNA or protein is also expressed in other normal tissues, such as liver and endocrine tissues. The potential toxicity and adverse effects of SORL1 inhibition are yet to be determined. Finally, since SORL1 acts as an intracellular sorting receptor of many proteins and directs them to their cellular locations, the inhibition of SORL1 may cause complex effects involving multiple downstream pathways. To develop effective approaches to target SORL1 in cancers, we will need to improve our knowledge of SORL1 functions in the context of specific cancer types. In addition to improving the methods of delivering the SORL1 antibody specifically to cancer cells, we also need to further evaluate the molecular, cellular, and in vivo effects of SORL1 inhibition.

## 5. Conclusions

We have provided in vitro and in vivo evidence to support the role of SORL1 in promoting carboplatin resistance of ovarian cancer cells. We further revealed that SORL1 interacts with EGFR and FGFR4, which may contribute to maintaining the levels of EGFR and FGFR4 proteins in ovarian cancer cells. Our findings also suggest that the downregulation or inhibition of SORL1 by shRNAs or a blocking antibody can inhibit SORL1 functions and downregulate EGFR and FGFR4 proteins. The inhibition of SORL1 has the potential to overcome carboplatin resistance in ovarian cancer cells.

## Data Availability

Data supporting reported results can be found at Dryad (an international open-access repository of research data).

## Supplementary Materials

The following supporting information can be downloaded at: https://www.mdpi.com/article/10.3390/cancers17020244/s1. Table S1: Information of antibodies; Table S2: Primers sequences; Table S3: Information of siRNA, shRNA, and plasmid; Table S4: Characteristics of 30 tumor samples from patients diagnosed with high-grade serous ovarian cancer. Table S5: Patient demographic table; Table S6: TCGA dataset of ovarian cancer.
