# Supplementary material for: SORL1-Mediated EGFR and FGFR4 Regulation Enhances Chemoresistance in Ovarian Cancer
Source: Cancers (Basel). 2025 Jan 13;17(2):244. doi: 10.3390/cancers17020244 (PMC11763764; doi:10.3390/cancers17020244)
Supplement: Supplementary file 1 [file cancers-17-00244-s001.zip › Table S1. RNA-QC.pdf]

Table S1. Information of antibodies

Sample ID	#	Nucleic Acid Conc. ng/ul	A260	A280	260/280	260/230
P1	1	96.74	2.419	1.28	1.89	2.55
S1	2	97.07	2.427	1.245	1.95	2.58
P2	3	104.4	2.61	1.35	1.93	1.61
S2	4	59.1	1.478	0.772	1.91	1.64
P3	5	40.5	1.012	0.586	1.73	1.27
S3	6	30.02	0.75	0.47	1.6	0.51
P4	7	96.12	2.403	1.227	1.96	2.33
S4	8	37.82	0.946	0.525	1.8	1.6
P5	9	42.64	1.066	0.563	1.89	2.96
S5	10	26.9	0.671	0.381	1.76	1.43
P6	11	118.5	2.963	1.551	1.91	2.46
S6	12	70.94	1.773	0.94	1.89	1.99
P7	13	70.1	1.753	0.985	1.78	0.56
S7	14	25.2	0.63	0.349	1.81	1.1
P8	15	53.5	1.338	0.774	1.73	0.76
S8	16	63.7	1.593	0.827	1.93	1.65
P9	17	25	0.624	0.38	1.64	0.83
S9	18	32.7	0.817	0.502	1.63	0.77
P10	19	21.2	0.529	0.33	1.61	0.63
S10	20	42.2	1.055	0.658	1.6	0.63
P11	21	37.8	0.944	0.495	1.91	1.04
S11	22	87.6	2.191	1.205	1.82	1.56
P12	23	112.8	2.819	1.516	1.86	1.88
S12	24	205.4	5.134	2.678	1.92	1.54
P13	25	94.4	2.359	1.229	1.92	1.76
S13	26	36.9	0.923	0.54	1.71	0.69
P14	27	59	1.474	0.762	1.94	1.6
S14	28	68.5	1.713	0.917	1.87	1.69
P15	29	117.8	2.945	1.575	1.87	1.71
S15	30	58.8	1.469	0.787	1.87	1.36
