# Supplementary material for: SORL1-Mediated EGFR and FGFR4 Regulation Enhances Chemoresistance in Ovarian Cancer
Source: Cancers (Basel). 2025 Jan 13;17(2):244. doi: 10.3390/cancers17020244 (PMC11763764; doi:10.3390/cancers17020244)
Supplement: Supplementary file 1 [file cancers-17-00244-s001.zip › Table S2. Antibodies.pdf]

Table S2. Antibody information

Target	Host species/ conjugation	Application	Dilution	Catalog number	Manufacturer
SORL1	Rabbit	Western blot, co-IP, PLA	1:1000	22592-1-AP	Proteintech
SORL1	Rabbit	Western blot	1:1000	79322	Cell Signaling Technology
SORL1	Mouse	Treatment	5-20 μg/mL	611860	BD Biosciences
GAPDH	Mouse/HRP	Western blot	1:10000	HRP-60004	Proteintech
EGFR	Mouse	Western blot, co-IP, PLA	1:1000	SC-373746	Santa Cruz Biotechnology
FGFR4	Mouse	Western blot, co-IP, PLA	1:500	SC-136988	Santa Cruz Biotechnology
Anti-mouse IgG	Horse/HRP	Western blot	1:1000	7076	Cell Signaling Technology
Anti-rabbit IgG	Goat/HRP	Western blot	1:1000	7074	Cell Signaling Technology
Mouse IgG	Mouse	PLA and treatment control	1:2000	68860	Cell Signaling Technology
