# Supplementary material for: SORL1-Mediated EGFR and FGFR4 Regulation Enhances Chemoresistance in Ovarian Cancer
Source: Cancers (Basel). 2025 Jan 13;17(2):244. doi: 10.3390/cancers17020244 (PMC11763764; doi:10.3390/cancers17020244)
Supplement: Supplementary file 1 [file cancers-17-00244-s001.zip › Table S3. Primers.pdf]

Table S3. Primer sequences.

Gene name	Primer sequences
ARPP19	Forward: 5′- AAGGAAACGGTTGCAGAAAGG -3′<br>Reverse: 5′- GGAGTGGGAATGTGGTCACC -3′
ATP6V1B2	Forward: 5′- AGTCAGTCGGAACTACCTCTC -3′<br>Reverse: 5′- CATCCGGTAAGGTCAAATGGAC -3′
KLF9	Forward: 5′- AGAGTGCATACAGGTGAACGG -3′<br>Reverse: 5′- AGTGTGGGTCCGGTAGTGG -3′
LITAF	Forward: 5′- ATGTCGGTTCCAGGACCTTAC -3′<br>Reverse: 5′- TACGAAGGAGGATTCATGCCC -3′
LSM2	Forward: 5′- CATCTGTGGAACCCTCCATTC -3′<br>Reverse: 5′- GCACGTATCGGACCACTGAG -3′
NABP1	Forward: 5′- CAGTGAACCCAACCCAGATTATC -3′<br>Reverse: 5′- GGTCCAAATGTACCTGTACCCAT -3′
OAZ1	Forward: 5′- GCCAAACGCATTAACTGGCG -3′<br>Reverse: 5′- TGTCCTCGCGGTTCTTGTG -3′
PPM1A	Forward: 5′- AGGGGCAGGGTAATGGGTT -3′<br>Reverse: 5′- GATCACAGCCGTATGTGCATC -3′
PPTC7	Forward: 5′- AGGACTTCCGTAAGGGCCTC -3′<br>Reverse: 5′- CGTTCACACGTCCGCATTAAA -3′
PQLC3 (SLC66A3)	Forward: 5′- GGCTGTGTAACTGGAGCAC -3′<br>Reverse: 5′- CCAGAAGTAAACTCGGAAGGCT -3′
PRS15	Forward: 5′- CCCGAGATGATCGGCCACTA -3′<br>Reverse: 5′- CCATGCTTTACGGGCTTGTAG -3′
RELL1	Forward: 5′- CCAGCAACGATACTGGGAATG -3′<br>Reverse: 5′- AAATGAGGACGCCAAAGAGAC -3′
SNRPD1	Forward: 5′- GAATTGAAGAACGGAACACAGGT -3′<br>Reverse: 5′- TCCACAAGTAGTGTATCCAGAGG -3′
SORL1	Forward: 5′- GAGCAGTGATGTGTACGTGTC -3′<br>Reverse: 5′- AGAACTGGGCGATAACAGCTT-3′
UBE2N	Forward: 5′- CAGATCCGCACAGTTCTGCTA -3′<br>Reverse: 5′- GGCTTCGTTGGTCTTCCACT -3′
GAPDH	Forward: 5′- AATGAAGGGGTCATTGATGG -3′<br>Reverse: 5′- AAGGTGAAGGTCGGAGTCAA -3′
