# Supplementary material for: SORL1-Mediated EGFR and FGFR4 Regulation Enhances Chemoresistance in Ovarian Cancer
Source: Cancers (Basel). 2025 Jan 13;17(2):244. doi: 10.3390/cancers17020244 (PMC11763764; doi:10.3390/cancers17020244)
Supplement: Supplementary file 1 [file cancers-17-00244-s001.zip › Table S4. siRNA-shRNA-plasmid.pdf]

Table S4. Characteristics of 30 tumor samples from patients diagnosed with high-grade serous ovarian cancer

**siRNA**:<br>Negative control esiRNA (SIC001)<br>SORL1-targeting esiRNAs (EHU003061)<br>*Purchased from Millipore-Sigma (The Woodlands, TX, USA)
**shRNA**:<br>Lentiviral negative control shRNA (sc-108080)<br>Lentiviral shRNA targeting SORL1 (sc-44375-V, a pool of 3~5 SORL1 specific shRNAs)<br>*Purchased from Santa Cruz Biotechnology, Dallas, TX, USA)
**Plasmid:**<br>pcDNA3.1(+)-SORL1 (with empty pcDNA3.1(+) vector as the negative control)<br>*Provided by Stephen M. Strittmatter (Reference: Levi M Smith, Mikhail A Kostylev, Suho Lee, Stephen M Strittmatter. Systematic and standardized comparison of reported amyloid-β receptors for sufficiency, affinity, and Alzheimer's disease relevance. J Biol Chem. 2019. 20;294(15):6042–6053. doi: 10.1074/jbc.RA118.006252.)
