# Supplementary material for: SORL1-Mediated EGFR and FGFR4 Regulation Enhances Chemoresistance in Ovarian Cancer
Source: Cancers (Basel). 2025 Jan 13;17(2):244. doi: 10.3390/cancers17020244 (PMC11763764; doi:10.3390/cancers17020244)
Supplement: Supplementary file 1 [file cancers-17-00244-s001.zip › Table S5. Patient Demographics.pdf]

Table S5. Patient demographic table

SORL1 expression in recurrent tumor vs. primary tumor	***SORL1 downregulated***	***SORL1 upregulated***
Patient number	2	13
Age at initial pathologic diagnosis		
30-40	1	0
40-60	0	10
>60	1	3
Histology		
Serous	2	13
Other	0	0
Tumor grade at initial diagnosis		
I	0	0
II	0	0
III	2	13
Tumor stage at initial diagnosis		
I	0	0
II	0	0
III	2	12
IV	0	1
Time of recurrent tumor collection (months post collection of primary tumor samples)		
<12 months	0	4
12-24 months	0	3
25-48 months	1	2
49-60 months	0	2
>60 months	1	2
Chemotherapy		
Platinum alone	0	0
Paclitaxel alone	0	0
Platinum-paclitaxel combination	2	13
