# Supplementary material for: SORL1-Mediated EGFR and FGFR4 Regulation Enhances Chemoresistance in Ovarian Cancer
Source: Cancers (Basel). 2025 Jan 13;17(2):244. doi: 10.3390/cancers17020244 (PMC11763764; doi:10.3390/cancers17020244)
Supplement: Supplementary file 1 [file cancers-17-00244-s001.zip › Table S6. SORL1 in TCGA-Ovarain cancer.pdf]

Table S6. TCGA dataset of ovarian cancer

Group	Accession number	Years post diagnosis	Status (0-survival; 1-deceased.)
SORL1-high	TCGA-57-1584	1.76	0
SORL1-high	TCGA-24-1103	4.51	1
SORL1-high	TCGA-36-1568	2.40	0
SORL1-high	TCGA-57-1993	2.09	0
SORL1-high	TCGA-23-1029	0.73	0
SORL1-high	TCGA-10-0938	1.74	1
SORL1-high	TCGA-24-1847	0.94	0
SORL1-high	TCGA-VG-A8LO	0.07	1
SORL1-high	TCGA-24-2254	4.76	1
SORL1-high	TCGA-61-1900	0.48	0
SORL1-high	TCGA-29-1776	0.99	0
SORL1-high	TCGA-24-2023	3.74	1
SORL1-high	TCGA-31-1946	2.52	0
SORL1-high	TCGA-24-1616	3.19	1
SORL1-high	TCGA-24-1549	4.72	1
SORL1-high	TCGA-24-2026	2.90	1
SORL1-high	TCGA-24-1563	3.98	1
SORL1-high	TCGA-24-1467	8.83	1
SORL1-high	TCGA-25-2400	3.50	1
SORL1-high	TCGA-24-1551	4.33	1
SORL1-high	TCGA-24-2027	9.14	1
SORL1-high	TCGA-09-1661	3.20	1
SORL1-high	TCGA-61-2101	4.62	1
SORL1-high	TCGA-25-1321	2.83	1
SORL1-high	TCGA-24-2289	5.61	1
SORL1-high	TCGA-59-2351	9.68	0
SORL1-high	TCGA-61-2097	5.05	0
SORL1-high	TCGA-24-1846	0.36	0
SORL1-high	TCGA-13-1511	4.52	1
SORL1-high	TCGA-25-1632	4.93	1
SORL1-high	TCGA-23-2078	7.29	0
SORL1-high	TCGA-61-2109	1.72	1
SORL1-high	TCGA-13-1489	6.99	1
SORL1-high	TCGA-29-1690	3.97	1
SORL1-high	TCGA-29-1688	6.58	1
SORL1-high	TCGA-23-1809	0.04	0
SORL1-high	TCGA-WR-A838	0.83	1
SORL1-high	TCGA-25-1631	0.02	1
SORL1-high	TCGA-25-1328	5.50	1
SORL1-high	TCGA-23-1119	10.83	0
SORL1-high	TCGA-25-1318	2.92	1
SORL1-high	TCGA-36-1571	1.90	1
SORL1-high	TCGA-61-1724	1.75	1
SORL1-high	TCGA-29-1695	3.37	1
SORL1-high	TCGA-OY-A56Q	1.58	0
SORL1-high	TCGA-09-1673	0.25	0
SORL1-high	TCGA-25-1326	3.42	1
SORL1-high	TCGA-13-1506	2.85	1
SORL1-high	TCGA-57-1586	1.86	0
SORL1-high	TCGA-61-1736	4.07	1
SORL1-high	TCGA-24-2036	5.33	1
SORL1-high	TCGA-23-1028	4.12	0
SORL1-high	TCGA-25-1313	2.25	1
SORL1-high	TCGA-24-1562	3.79	1
SORL1-high	TCGA-24-1422	0.06	1
SORL1-high	TCGA-24-1413	0.53	0
SORL1-high	TCGA-24-1565	0.85	1
SORL1-high	TCGA-24-1464	1.04	1
SORL1-high	TCGA-36-1574	1.88	0
SORL1-high	TCGA-25-1627	1.08	1
SORL1-high	TCGA-30-1862	0.51	1
SORL1-high	TCGA-25-1630	3.18	1
SORL1-high	TCGA-29-1785	3.02	1

SORL1-high	TCGA-30-1857	0.02	1
SORL1-high	TCGA-24-1844	0.31	0
SORL1-high	TCGA-61-1918	1.31	1
SORL1-high	TCGA-29-1697	2.60	1
SORL1-high	TCGA-24-2290	3.02	1
SORL1-high	TCGA-09-2051	5.26	0
SORL1-high	TCGA-04-1362	3.69	1
SORL1-high	TCGA-09-1668	4.61	0
SORL1-high	TCGA-24-1604	7.36	1
SORL1-high	TCGA-25-1322	0.25	1
SORL1-high	TCGA-25-1312	0.08	1
SORL1-high	TCGA-25-1315	4.34	1
SORL1-high	TCGA-24-1845	0.32	0
SORL1-high	TCGA-23-1118	7.17	0
SORL1-high	TCGA-23-1120	0.36	0
SORL1-high	TCGA-25-1635	4.34	1
SORL1-high	TCGA-25-2399	1.67	1
SORL1-high	TCGA-59-2354	2.87	1
SORL1-high	TCGA-61-2088	0.40	0
SORL1-high	TCGA-24-1546	5.36	1
SORL1-high	TCGA-57-1994	2.08	0
SORL1-high	TCGA-24-1416	0.53	0
SORL1-high	TCGA-23-1023	3.38	0
SORL1-high	TCGA-24-1105	3.95	1
SORL1-high	TCGA-59-2355	0.18	1
SORL1-high	TCGA-04-1361	2.71	0
SORL1-high	TCGA-09-2045	2.93	1
SORL1-high	TCGA-31-1959	0.18	0
SORL1-high	TCGA-24-2293	1.39	1
SORL1-high	TCGA-24-1431	1.55	1
SORL1-high	TCGA-61-1995	0.17	0
SORL1-high	TCGA-13-A5FT	5.87	0
SORL1-high	TCGA-59-2363	0.45	0
SORL1-high	TCGA-04-1651	3.02	1
SORL1-high	TCGA-24-1104	5.30	1
SORL1-high	TCGA-29-1694	3.25	1
SORL1-high	TCGA-09-1670	1.50	0
SORL1-high	TCGA-09-0367	1.50	1
SORL1-high	TCGA-20-1686	0.24	0
SORL1-high	TCGA-24-1417	0.65	0
SORL1-high	TCGA-04-1542	7.02	1
SORL1-high	TCGA-24-1469	0.76	0
SORL1-high	TCGA-61-2012	2.55	0
SORL1-high	TCGA-24-1423	0.52	0
SORL1-high	TCGA-59-A5PD	1.71	1
SORL1-high	TCGA-24-1557	3.32	1
SORL1-high	TCGA-24-1550	3.42	1
SORL1-high	TCGA-24-1434	1.56	1
SORL1-high	TCGA-20-1687	0.22	0
SORL1-high	TCGA-24-2262	0.03	1
SORL1-high	TCGA-09-1662	7.44	1
SORL1-high	TCGA-13-0797	5.81	0
SORL1-high	TCGA-61-2008	2.55	0
SORL1-high	TCGA-29-2425	5.42	0
SORL1-high	TCGA-25-1323	1.08	1
SORL1-high	TCGA-24-1553	4.84	1
SORL1-high	TCGA-13-0714	0.52	1
SORL1-high	TCGA-31-1953	0.56	0
SORL1-high	TCGA-29-1705	1.52	1
SORL1-high	TCGA-13-0883	5.75	1
SORL1-high	TCGA-25-2409	2.25	1
SORL1-high	TCGA-13-0920	4.07	1
SORL1-high	TCGA-04-1519	0.07	0
SORL1-high	TCGA-13-0804	2.94	1
SORL1-high	TCGA-13-0885	9.28	0
SORL1-high	TCGA-04-1648	2.39	1

SORL1-high	TCGA-23-1114	5.72	1
SORL1-high	TCGA-61-1738	2.98	1
SORL1-high	TCGA-13-0887	5.56	1
SORL1-high	TCGA-24-0966	0.64	0
SORL1-high	TCGA-36-1569	2.42	0
SORL1-high	TCGA-25-1626	1.42	1
SORL1-high	TCGA-29-1701	1.41	1
SORL1-high	TCGA-61-2098	5.46	0
SORL1-high	TCGA-09-1665	3.47	1
SORL1-high	TCGA-36-1576	2.51	0
SORL1-high	TCGA-57-1585	0.15	1
SORL1-high	TCGA-24-1419	0.65	0
SORL1-high	TCGA-13-0726	2.60	1
SORL1-high	TCGA-24-1603	7.51	1
SORL1-high	TCGA-36-1570	1.79	0
SORL1-high	TCGA-09-0366	4.81	1
SORL1-high	TCGA-23-1022	4.14	1
SORL1-high	TCGA-31-1951	1.87	0
SORL1-high	TCGA-57-1582	2.00	1
SORL1-high	TCGA-24-1471	0.10	0
SORL1-high	TCGA-20-1682	2.29	0
SORL1-high	TCGA-31-1944	3.80	0
SORL1-high	TCGA-36-1577	2.15	0
SORL1-high	TCGA-25-2391	4.09	1
SORL1-high	TCGA-24-2297	4.65	1
SORL1-high	TCGA-25-1317	0.17	1
SORL1-high	TCGA-13-0766	4.73	1
SORL1-high	TCGA-59-2350	1.86	1
SORL1-high	TCGA-13-1477	4.55	1
SORL1-high	TCGA-13-0795	1.70	1
SORL1-high	TCGA-10-0933	1.22	1
SORL1-high	TCGA-04-1530	9.92	1
SORL1-high	TCGA-24-1567	1.44	1
SORL1-high	TCGA-24-1470	0.29	0
SORL1-high	TCGA-13-1498	5.51	1
SORL1-high	TCGA-23-1027	2.67	1
SORL1-high	TCGA-10-0937	1.67	1
SORL1-high	TCGA-29-2427	5.21	0
SORL1-high	TCGA-59-2352	0.78	1
SORL1-high	TCGA-23-1111	0.27	0
SORL1-high	TCGA-13-0897	5.98	1
SORL1-high	TCGA-5X-AA5U	0.99	0
SORL1-high	TCGA-61-2000	1.21	0
SORL1-high	TCGA-30-1718	4.33	1
SORL1-high	TCGA-13-1487	1.87	1
SORL1-high	TCGA-29-1763	5.57	0
SORL1-high	TCGA-61-1998	0.46	0
SORL1-high	TCGA-25-1870	1.25	1
SORL1-high	TCGA-31-1950	1.56	0
SORL1-high	TCGA-04-1365	6.38	0
SORL1-high	TCGA-30-1861	2.90	1
SORL1-high	TCGA-24-1430	2.36	1
SORL1-high	TCGA-13-1505	5.47	0
SORL1-high	TCGA-04-1332	3.42	1
SORL1-high	TCGA-13-1403	6.42	1
SORL1-high	TCGA-24-1560	3.67	1
SORL1-high	TCGA-61-2110	3.71	1
SORL1-high	TCGA-13-0908	5.05	1
SORL1-high	TCGA-29-1691	4.03	1
SORL1-high	TCGA-13-1405	2.38	1
SORL1-high	TCGA-04-1350	5.33	1
SORL1-high	TCGA-23-1107	0.02	1
SORL1-high	TCGA-10-0931	2.74	1
SORL1-high	TCGA-13-0800	7.29	0
SORL1-high	TCGA-24-1474	1.85	1
SORL1-high	TCGA-61-1737	3.74	0

SORL1-high	TCGA-24-2271	2.64	1
SORL1-high	TCGA-29-A5NZ	2.98	1
SORL1-high	TCGA-24-1924	2.52	1
SORL1-high	TCGA-61-1728	2.32	0
SORL1-high	TCGA-23-1021	3.96	1
SORL1-high	TCGA-29-1710	2.61	1
SORL1-high	TCGA-61-1741	2.81	1
SORL1-high	TCGA-24-2288	0.07	1
SORL1-high	TCGA-29-1703	4.97	1
SORL1-high	TCGA-61-2002	1.50	0
SORL1-high	TCGA-23-1024	1.28	0
SORL1-high	TCGA-24-2033	1.54	1
SORL1-high	TCGA-29-1784	0.45	0
SORL1-high	TCGA-24-2298	4.44	1
SORL1-low	TCGA-24-2035	2.35	1
SORL1-low	TCGA-13-1488	5.90	1
SORL1-low	TCGA-31-1956	3.68	0
SORL1-low	TCGA-25-2404	2.42	1
SORL1-low	TCGA-24-2281	3.72	0
SORL1-low	TCGA-61-1907	2.61	0
SORL1-low	TCGA-13-0886	12.78	0
SORL1-low	TCGA-24-1923	1.89	1
SORL1-low	TCGA-29-1783	0.60	0
SORL1-low	TCGA-61-2111	10.48	0
SORL1-low	TCGA-09-2053	3.31	0
SORL1-low	TCGA-13-0924	7.16	0
SORL1-low	TCGA-25-1628	1.72	1
SORL1-low	TCGA-13-0725	1.03	1
SORL1-low	TCGA-24-1427	0.40	0
SORL1-low	TCGA-24-2024	4.85	1
SORL1-low	TCGA-24-1424	0.50	0
SORL1-low	TCGA-04-1343	0.99	1
SORL1-low	TCGA-29-1696	2.83	1
SORL1-low	TCGA-23-1030	2.43	0
SORL1-low	TCGA-24-1930	6.76	1
SORL1-low	TCGA-23-1122	3.26	1
SORL1-low	TCGA-25-1623	1.55	1
SORL1-low	TCGA-13-0762	9.32	0
SORL1-low	TCGA-24-2261	0.07	1
SORL1-low	TCGA-23-1026	2.24	0
SORL1-low	TCGA-25-1319	5.42	1
SORL1-low	TCGA-24-2267	3.96	1
SORL1-low	TCGA-61-2113	1.85	1
SORL1-low	TCGA-25-1634	2.99	1
SORL1-low	TCGA-09-1669	2.54	0
SORL1-low	TCGA-36-1580	2.02	1
SORL1-low	TCGA-29-1693	8.48	0
SORL1-low	TCGA-29-1761	1.45	1
SORL1-low	TCGA-25-1316	3.50	1
SORL1-low	TCGA-29-2414	7.18	1
SORL1-low	TCGA-13-1411	1.45	1
SORL1-low	TCGA-24-2038	3.71	1
SORL1-low	TCGA-24-1558	1.63	1
SORL1-low	TCGA-13-1483	2.45	1
SORL1-low	TCGA-29-2428	3.76	0
SORL1-low	TCGA-23-2084	4.15	1
SORL1-low	TCGA-61-2104	6.41	0
SORL1-low	TCGA-30-1714	3.17	1
SORL1-low	TCGA-61-1733	2.65	0
SORL1-low	TCGA-24-1428	1.45	0
SORL1-low	TCGA-10-0928	1.54	1
SORL1-low	TCGA-25-2401	0.25	1
SORL1-low	TCGA-25-1320	3.16	1
SORL1-low	TCGA-24-1418	0.67	0
SORL1-low	TCGA-04-1514	4.71	1
SORL1-low	TCGA-24-1843	0.29	0

SORL1-low	TCGA-13-1404	6.76	0
SORL1-low	TCGA-30-1860	3.74	1
SORL1-low	TCGA-61-2003	0.33	0
SORL1-low	TCGA-24-1544	2.25	1
SORL1-low	TCGA-23-2077	9.66	0
SORL1-low	TCGA-23-1116	1.62	1
SORL1-low	TCGA-25-2396	0.25	1
SORL1-low	TCGA-13-1409	4.77	1
SORL1-low	TCGA-29-1770	2.03	0
SORL1-low	TCGA-04-1341	0.09	0
SORL1-low	TCGA-04-1347	5.26	0
SORL1-low	TCGA-13-1509	6.68	0
SORL1-low	TCGA-13-0905	10.26	0
SORL1-low	TCGA-23-1123	2.79	1
SORL1-low	TCGA-13-0765	3.81	1
SORL1-low	TCGA-61-1725	2.62	0
SORL1-low	TCGA-61-1910	3.09	0
SORL1-low	TCGA-04-1331	3.66	1
SORL1-low	TCGA-24-1842	0.69	0
SORL1-low	TCGA-29-1769	1.92	0
SORL1-low	TCGA-13-1496	0.35	1
SORL1-low	TCGA-29-1774	1.44	0
SORL1-low	TCGA-13-1407	6.94	0
SORL1-low	TCGA-25-2392	0.08	1
SORL1-low	TCGA-29-1711	2.88	0
SORL1-low	TCGA-13-0901	1.61	0
SORL1-low	TCGA-13-1512	1.21	0
SORL1-low	TCGA-29-1762	7.22	1
SORL1-low	TCGA-24-0979	3.46	1
SORL1-low	TCGA-13-2060	6.49	0
SORL1-low	TCGA-61-2009	3.32	0
SORL1-low	TCGA-61-1911	3.54	0
SORL1-low	TCGA-09-1659	0.83	1
SORL1-low	TCGA-61-1919	3.18	1
SORL1-low	TCGA-23-1110	4.54	0
SORL1-low	TCGA-13-0724	0.23	1
SORL1-low	TCGA-13-1485	1.72	1
SORL1-low	TCGA-29-1766	3.28	1
SORL1-low	TCGA-61-1914	4.72	0
SORL1-low	TCGA-25-2398	3.75	1
SORL1-low	TCGA-24-1850	0.46	0
SORL1-low	TCGA-59-2348	15.02	0
SORL1-low	TCGA-13-1501	3.60	1
SORL1-low	TCGA-25-2393	3.17	1
SORL1-low	TCGA-13-0923	6.98	0
SORL1-low	TCGA-24-1928	0.92	1
SORL1-low	TCGA-25-2042	1.08	1
SORL1-low	TCGA-57-1583	0.95	1
SORL1-low	TCGA-29-1778	1.24	0
SORL1-low	TCGA-13-0906	9.92	0
SORL1-low	TCGA-13-0888	7.70	1
SORL1-low	TCGA-23-1109	4.28	1
SORL1-low	TCGA-61-2092	4.31	0
SORL1-low	TCGA-13-0900	11.17	0
SORL1-low	TCGA-13-1492	10.46	1
SORL1-low	TCGA-30-1853	3.02	1
SORL1-low	TCGA-24-1426	0.45	0
SORL1-low	TCGA-24-0982	1.86	1
SORL1-low	TCGA-13-1499	9.59	0
SORL1-low	TCGA-24-1435	3.63	1
SORL1-low	TCGA-13-1495	7.53	1
SORL1-low	TCGA-13-0884	8.93	1
SORL1-low	TCGA-30-1866	3.05	1
SORL1-low	TCGA-25-1329	1.25	1
SORL1-low	TCGA-09-1666	4.80	0
SORL1-low	TCGA-30-1891	2.50	1

SORL1-low	TCGA-36-1581	2.06	0
SORL1-low	TCGA-09-2056	1.04	0
SORL1-low	TCGA-09-2054	1.75	1
SORL1-low	TCGA-24-0968	1.64	1
SORL1-low	TCGA-13-1497	10.61	0
SORL1-low	TCGA-04-1655	3.78	1
SORL1-low	TCGA-13-0893	3.61	1
SORL1-low	TCGA-10-0936	3.08	1
SORL1-low	TCGA-20-0987	1.92	1
SORL1-low	TCGA-04-1338	3.88	0
SORL1-low	TCGA-29-1768	2.61	1
SORL1-low	TCGA-61-1721	0.93	0
SORL1-low	TCGA-04-1536	2.42	1
SORL1-low	TCGA-09-2044	0.51	0
SORL1-low	TCGA-24-1425	0.50	0
SORL1-low	TCGA-13-1410	6.75	0
SORL1-low	TCGA-29-1777	1.02	0
SORL1-low	TCGA-09-0364	2.43	1
SORL1-low	TCGA-09-1667	5.16	0
SORL1-low	TCGA-30-1892	4.07	1
SORL1-low	TCGA-25-1633	5.18	1
SORL1-low	TCGA-04-1364	2.81	1
SORL1-low	TCGA-10-0927	6.82	1
SORL1-low	TCGA-04-1356	4.11	1
SORL1-low	TCGA-25-1877	2.00	1
SORL1-low	TCGA-13-0911	3.71	1
SORL1-low	TCGA-13-1510	3.72	1
SORL1-low	TCGA-13-1408	4.60	1
SORL1-low	TCGA-09-0369	2.96	1
SORL1-low	TCGA-13-0720	3.71	1
SORL1-low	TCGA-23-1113	2.60	1
SORL1-low	TCGA-20-0991	2.18	0
SORL1-low	TCGA-13-0727	1.27	1
SORL1-low	TCGA-13-0768	4.89	1
SORL1-low	TCGA-13-0730	1.48	1
SORL1-low	TCGA-13-0916	4.89	0
SORL1-low	TCGA-24-1552	3.45	1
SORL1-low	TCGA-24-0970	0.97	1
SORL1-low	TCGA-09-2048	0.38	1
SORL1-low	TCGA-13-0891	8.57	1
SORL1-low	TCGA-61-2102	0.54	1
SORL1-low	TCGA-24-2280	5.87	0
SORL1-low	TCGA-13-1507	5.46	1
SORL1-low	TCGA-29-1781	0.70	0
SORL1-low	TCGA-20-1683	2.12	0
SORL1-low	TCGA-24-2020	12.67	1
